# Supplementary material for: Conjugation dynamics depend on both the plasmid acquisition cost and the fitness cost
Source: Mol Syst Biol. 2021 Mar 1;17(3):e9913. doi: 10.15252/msb.20209913 (PMC7919528; doi:10.15252/msb.20209913)
Supplement: Supplementary file 1 — Appendix [file MSB-17-e9913-s001.pdf]

Appendix for:

Conjugation dynamics depend on both the plasmid acquisition cost and the fitness cost

Hannah Prensky[1], Angela Gomez-Simmonds[2], Anne-Catrin Uhlemann[2], and Allison J. Lopatkin[1,3,4,*]

[1]Department of Biology, Barnard College; New York, NY 10027; USA

[2]Department of Medicine, Division of Infectious Diseases, Columbia University Medical Center, New York, NY 10032

[3]Department Ecology, Evolution, and Environmental Biology, Columbia University; New York, NY 10027; USA

[4]Data Science Institute, Columbia University; New York, NY 10027; USA

[*]Corresponding author: alopatkin@barnard.edu

Table of Contents

Appendix Supplementary Methods 3
Appendix Figure S1: RP4 validation 5
Appendix Figure S2: Effects of acquisition cost are independent of method used to quantify RP4 growth parameters 6
Appendix Figure S3: Time to threshold reliably distinguishes diverse acquisition costs. 7
Appendix Figure S4: Time to threshold metric validation 8
Appendix Figure S5: Fitness costs for other plasmids and experimental conditions. 9
Appendix Figure S6: Base conjugation model 10
Appendix Figure S7: Fitted acquisition costs for diverse plasmids 12
Appendix Figure S8: Updated persistence criteria 13
Appendix Figure S9: Growth rate ratio cutoff validation 14
Appendix Figure S10: Data reproducibility 15
Appendix Table S1: Strains and plasmids used in this study 16
Appendix Table S2: Growth rate and lag time statistics using one-way ANOVA with Bonferroni correction 18
Appendix Table S3: P-values for acquisition costs 19
Appendix Table S4: Model parameters and variable values 20
References 21

Appendix Supplementary Methods

Mathematical modeling

The full model is described in Eq. 1-3 is based on our previously published model consisting of two populations where one carries a plasmid (S^1) and the other does not (S^0) (Eq. S1-2):

1. $\frac{dS^0}{dt} = \alpha\mu S^0(1 - S^0 - S^1) - \eta S^0 S^A + \kappa S^1 - DS^0$

2. $\frac{dS^1}{dt} = \mu S^1(1 - S^0 - S^1) + \eta S^0 S^1 - \kappa S^1 - DS^1$

Here, S^1 and S^0 correspond to the population density normalized with respect to its carrying capacity. α is the plasmid fitness cost, μ is the growth rate of the plasmid-carrying population, η is the conjugation efficiency, κ is the plasmid loss rate, and D is the dilution rate. From this, and based on our experimental data, we considered the scenario where upon conjugation, cells first enter a specific physiological state upon which they must adapt to the new metabolic burden; these *de novo* transconjugants institute a delay on the formation of growing adapted cells. This is described in Eq. S3-5, which consists of all three populations S^D, S^A, and S^0, corresponding to *de novo* transconjugants, adapted transconjugants, and plasmid-free cells, respectively:

3. $\frac{dS^0}{dt} = \alpha\mu S^0(1 - S^0 - S^A - S^D) - \eta S^0 S^A + \kappa S^A - DS^0$

4. $\frac{dS^D}{dt} = \rho\mu S^D(1 - S^0 - S^A - S^D) + \eta S^0 S^A - \beta S^D - DS^D$

5. $\frac{dS^A}{dt} = \mu S^A(1 - S^0 - S^A - S^D) + \beta S^D - \kappa S^A - DS^A$

In addition to those parameters from the base model, here we define ρ to be the scalar describing *de novo* population growth rate relative to the fully adapted population, and β to be the transition rate between the two plasmid-carrying populations. We further assume that the *de novo* population does not appreciably contribute to itself through conjugation, nor to S^0 through plasmid loss. These limiting assumptions do not change the qualitative behavior of the system.

All data fitting of acquisition cost experiments was done assuming that the initial conditions of all three populations was 0, x, and 0, for S^0, S^D, and S^A respectively, where x corresponds to the number of cells quantified via colony forming units (CFU). Only ρ and β were fit by optimizing the minimum distance between our model and data using the custom MATLAB scripts and the function fminsearch. To calculate the observed growth rate (e.g., in Fig 4C), we calculate the weighted average of the total plasmid-carrying population (e.g., S^D+S^A) as described in Eq. S6:

6. $$\mu_{obs} = \frac{\rho\mu S^D + \mu S^A}{S^D + S^A}$$

To numerically determine if μ and μ_{obs} were different, we set the threshold for μ_{obs}/μ to 98%, which was sufficiently high to capture the quantitative and qualitative trends but not too high to result in artifacts; this threshold does not make any qualitative difference to our interpretation of the heat map (Appendix Fig S7).

To investigate whether the contribution of conjugation through *de novo* transconjugants would significantly impact the dynamics, we further expanded our full model to account for additional conjugation between S^D and S^0. In particular, *de novo* transconjugants could arise due to conjugation between S^0 and S^A at a specific rate constant η_1 and between S^0 and S^D at the rate η_2:

7. $$\frac{dS^0}{dt} = \alpha\mu S^0(1 - S^0 - S^A - S^D) - \eta_1 S^0 S^A - \eta_2 S^0 S^D + \kappa S^A - DS^0$$

8. $$\frac{dS^D}{dt} = \rho\mu S^D(1 - S^0 - S^A - S^D) + \eta_1 S^0 S^A + \eta_2 S^0 S^D - \beta S^D - DS^D$$

9. $$\frac{dS^A}{dt} = \mu S^A(1 - S^0 - S^A - S^D) + \beta S^D - \kappa S^A - DS^A$$

Using this model, we investigated whether different values of η_2 would significantly influence the observed short and long-term outcomes of emergent transconjugants. Results indicated that conjugation of *de novo* transconjugants did not significantly alter the long-term dynamics, and only moderately impacted the short-term effects (Appendix Fig S6C, top). Moreover, the predictability of the model with *de novo* conjugation did not significantly change (Appendix Fig S6C, bottom). Thus, the original full model was used for all primary simulations.

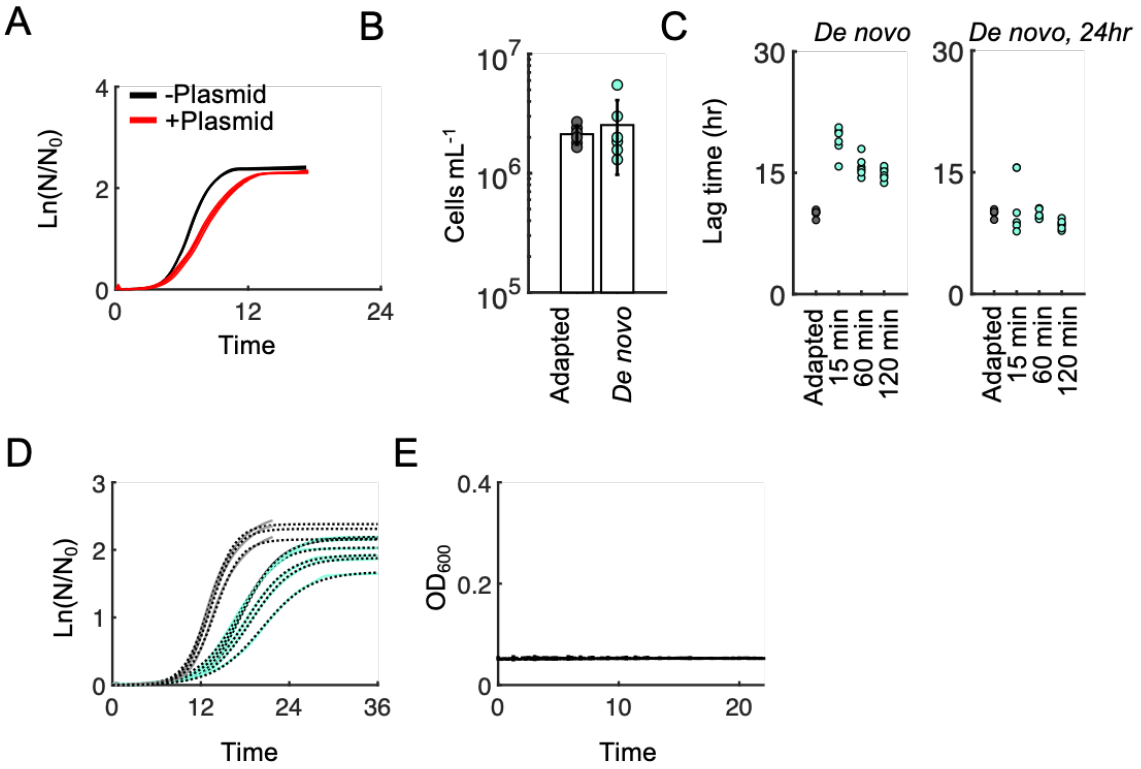

Appendix Figure S1: RP4 validation

A) Representative growth curves for RP4 (red) and its plasmid-free counterpart (black); cells are diluted 10,000X in M9CA and grown over 16 hours (x-axis). Y-axis is log-transformed normalized optical density (OD_{600}).
B) Initial number of cells from each condition in Fig 1C as determined by CFU count.
C) Non-normalized lag times, raw data from Fig 1E-F.
D) *De novo* T with R/D competition (same as in Fig 1C) with 1,000X dilution into the background media. Gray is adapted T and blue is *de novo* T; black lines are fitted with Baranyi equations.
E) R and D cells do not grow when diluted 1,000X into media containing Spec and Kan.

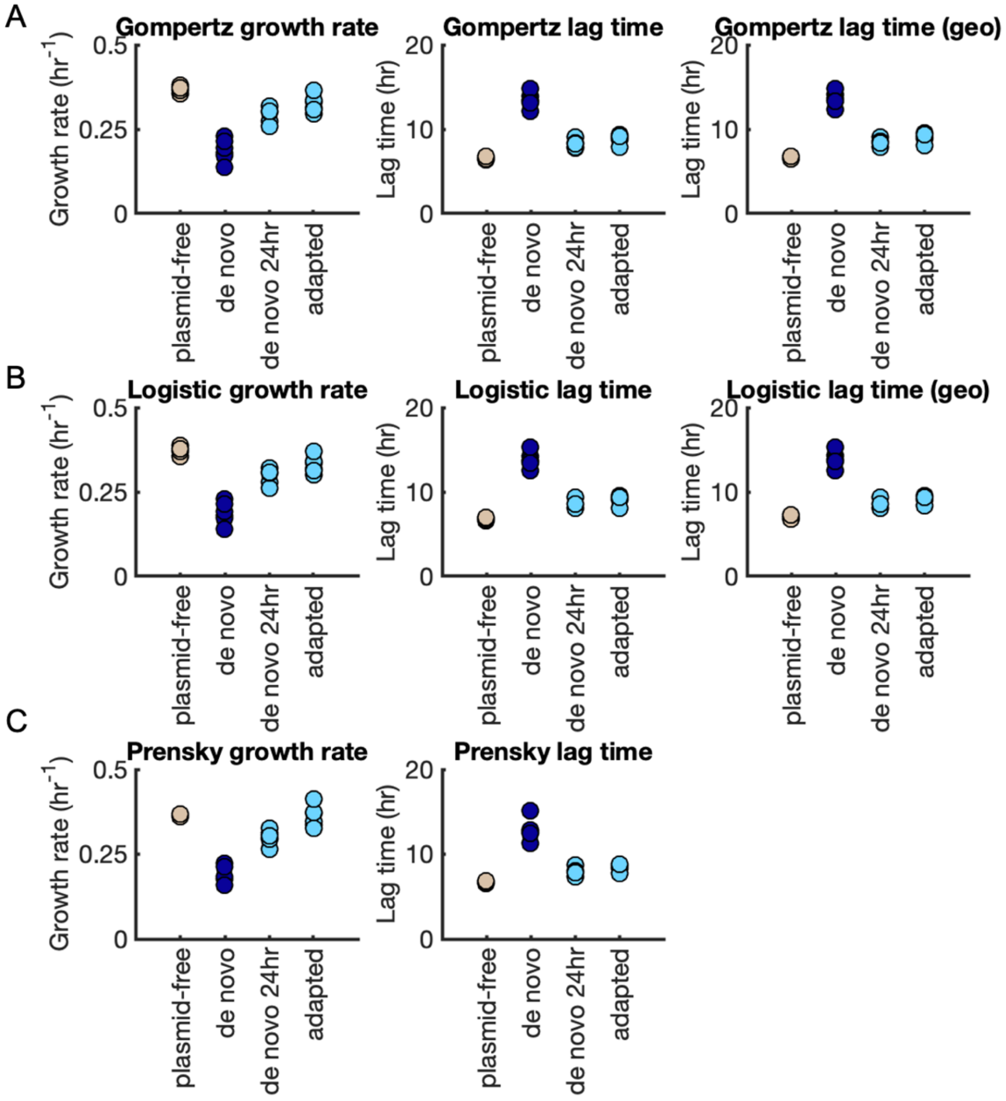

Appendix Figure S2: Effects of acquisition cost are independent of method used to quantify RP4 growth parameters

A) Gompertz growth rate (left), lag time (middle), and geometric lag time (right) for plasmid-free, *de novo* T after 1 hour, *de novo* T after 24 hours, and adapted T cells. Growth rate is statistically less for *de novo* cells (1 hr) than for plasmid-free and adapted cells (Appendix Table S2). Lag time and geometric lag time are statistically greater for *de novo* cells (1 hr) than for plasmid-free and adapted cells (Appendix Table S2).

B) Logistic growth rate (left), lag time (middle), and geometric lag time (right) for plasmid-free, *de novo* T after 1 hour, *de novo* T after 24 hours, and adapted T cells. Growth rate is statistically less for *de novo* cells (1 hr) than for plasmid-free and adapted cells (Appendix Table S2). Lag time and geometric lag time are statistically greater for *de novo* cells (1 hr) than for plasmid-free and adapted cells (Appendix Table S2).

C) Prensky growth rate (left) and lag time (right) for plasmid-free, *de novo* T after 1 hour, *de novo* T after 24 hours, and adapted T cells. Growth rate is statistically less for *de novo* cells (1 hr) than for plasmid-free and adapted cells (Appendix Table S2). Lag time is statistically greater for *de novo* cells (1 hr) than for plasmid-free and adapted cells (Appendix Table S2).

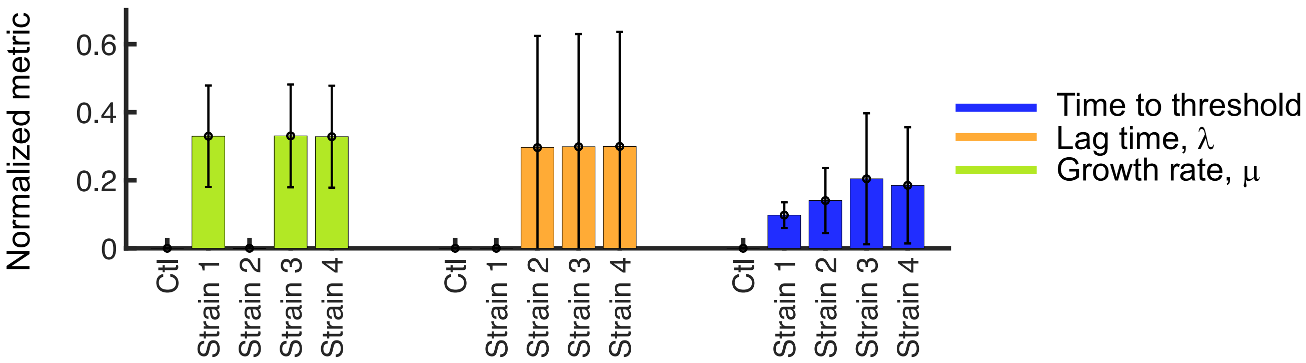

Appendix Figure S3: Time to threshold reliably distinguishes diverse acquisition costs.

Strains 1-4 correspond to variant populations (i.e., parameter sets) that differ from the baseline (i.e., Ctl) by randomizing specific parameter(s) while keeping all others constant: growth rate, lag time, both growth rate and lag time, and growth rate/lag time/initial density, respectively. For each strain, 1000 random variants were generated, and each calculated metric was normalized to the corresponding Ctl strain metric as follows (e.g. growth rate): $\mu_{norm} = \left|\log_{10}\frac{\mu_{variant}}{\mu_{Ctl}}\right|$. Therefore, any variant metric that differed from the corresponding control strain metric mapped to a positive value; any variant metric identical to the control strain mapped to 0. Average normalized metrics are shown for each of Strains 1-4 (growth rate, lag time, and time to threshold shown in green, orange, and blue, respectively); a given metric is capable of distinguishing between a variant and the control only when the average normalized value is significantly greater than 0. Only time to threshold, as opposed to growth rate or lag time, was able to distinguish all types of variants (Strains 1-4, i.e., different modes of acquisition cost) from the control (i.e., fully adapted) strain.

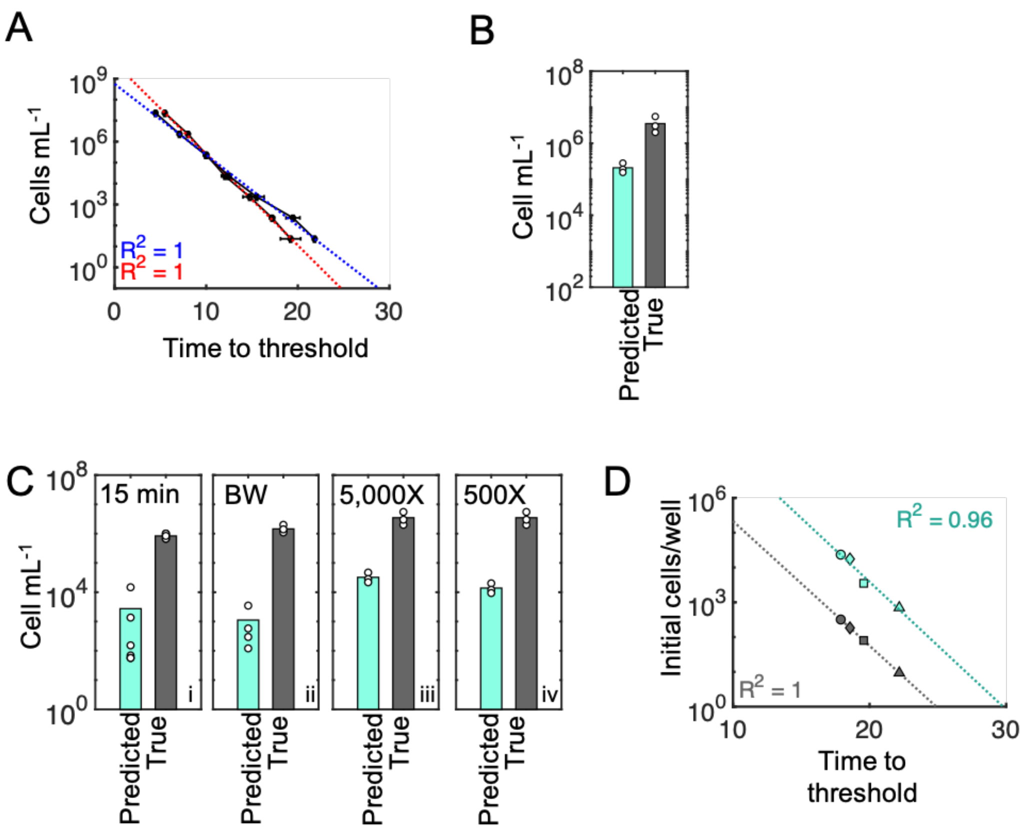

Appendix Figure S4: Time to threshold metric validation

A) Original standard curve (e.g., Fig 2C) shown in blue (no R and D in background), and new standard curve where R and D are diluted 1000X into the growth media shown in red. R^2 values are from least-squares linear regression.

B) The updated standard curve (in red) was used to calculate T_{pred}; it is still statistically less than true T_0, demonstrating that the background presence of R and D likely had minimal effect.

C) True and predicted cell density of RP4 under the specified experimental conditions, from left to right: 15-minute conjugation time window (i), recipient strain BW25113 (ii), 5,000X dilution into out-growth (iii), and 500X dilution into out-growth (iv).

D) Following the 60-minute conjugation, cells were diluted 150X, 500X, 1000X, or 5000X (circle, diamond, square, and triangle, respectively).

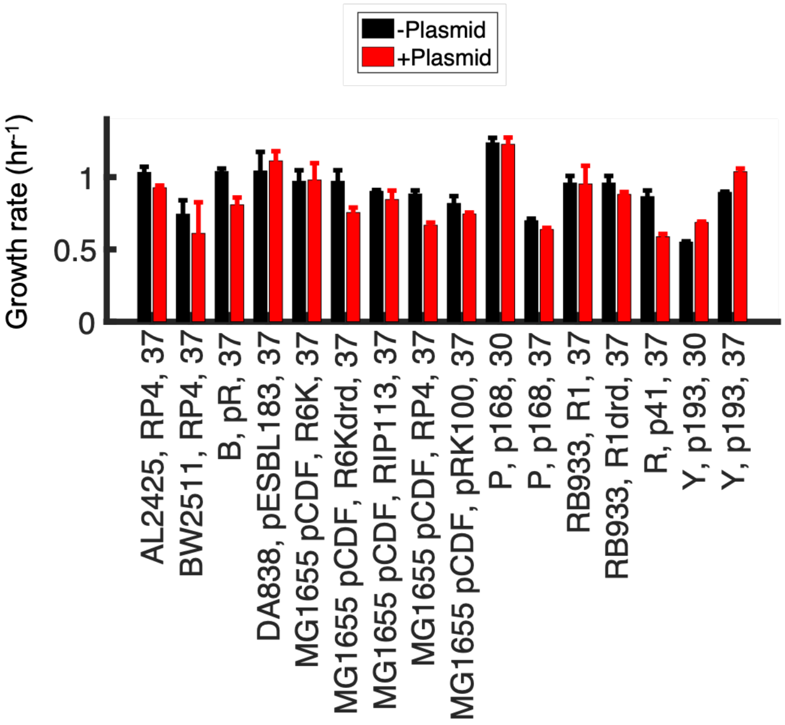

Appendix Figure S5: Fitness costs for other plasmids and experimental conditions.

Growth rates are directly compared between the plasmid-carrying (red) and plasmid-free counterparts. Error bars represent the averages of three or more biological replicates. X-labels refer to [strain, plasmid, temperature] respectively.

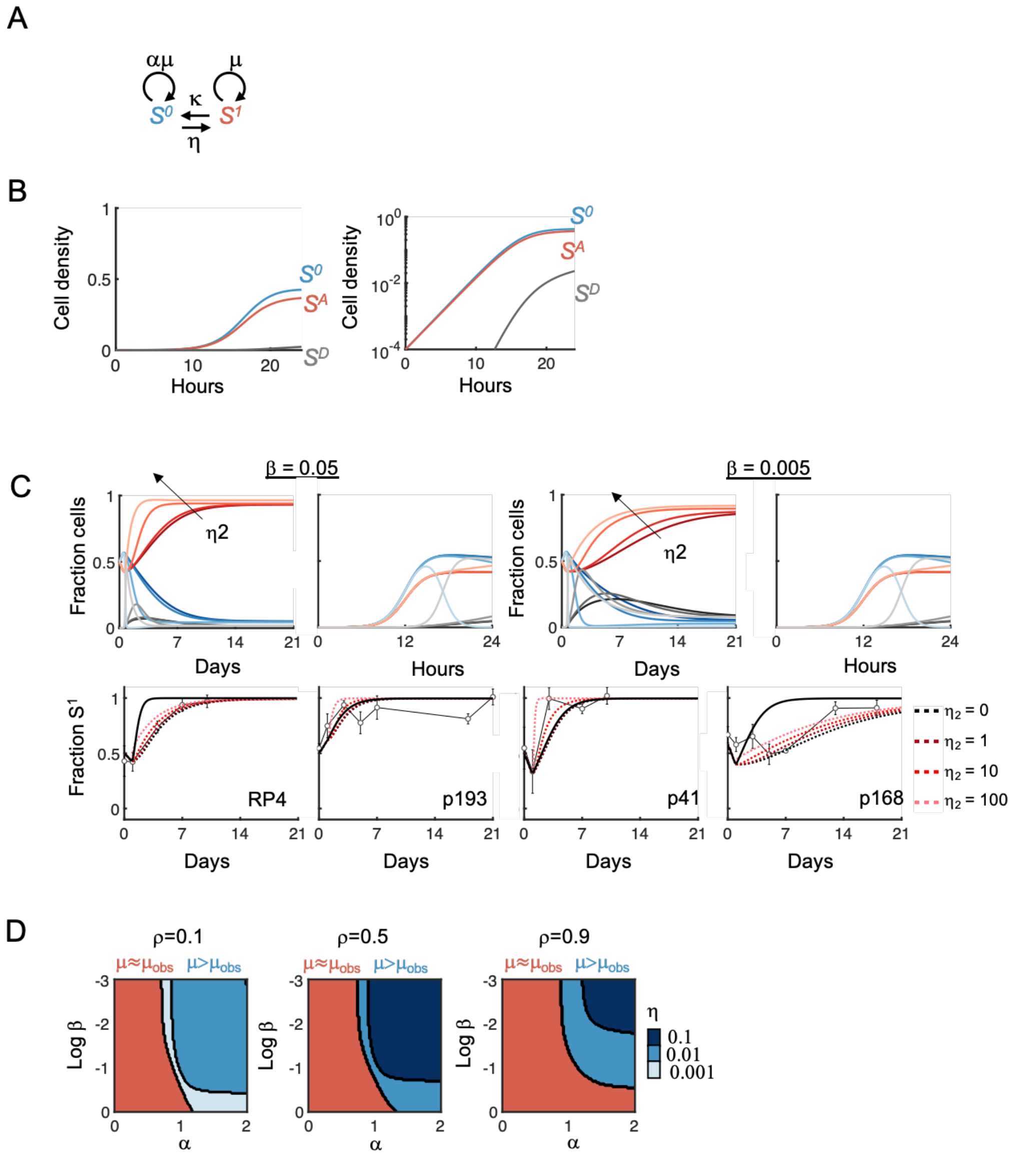

Appendix Figure S6: Base conjugation model

A) The plasmid-free population (S^0) acquires the plasmid at a rate η, becoming S^1, the plasmid carrying population. S^1 can lose the plasmid at a rate κ corresponding to the plasmid segregation error rate. Each population grows at a rate relative to the other, where μ is the growth rate of S^1 and $\alpha\mu$ is the growth rate of S^2.

B) Short-term temporal dynamics of S^A, S^D, and S^0 in red, blue, and gray respectively from the main model (Fig 4A). X-axis is time over 24 hours and y-axis is the total cell density of each population. Simulations are shown on linear (left) and log-scaled (right) y-axes.

C) *Top row:* Simulations were implemented according to Appendix equations S8-S10. Results are shown for high (top row, left) and low (top row, right) β values, as this parameter most drastically impacted the observed dynamics. Temporal dynamics of S^A, S^D, and S^0 in red, blue, and gray are shown, respectively; shading indicates ranging η_2 from 0 to 100X above the basal level). Higher acquisition

cost (lower β) increases the impact of conjugation from S^D. *Bottom row:* Impact of S^D conjugation on long-term dynamics for the same range of η_2 as in the top row.

D) Same simulations as in Fig 5C with increasing values of ρ from left to right 0.1, 0.5 and 0.9. Red indicates $\mu_{obs}/\mu>0.98$, and shades of blue indicate $\mu_{obs}/\mu<0.98$. Light to dark blue shades correspond to η equal to 0.001, 0.01, and 0.1 respectively.

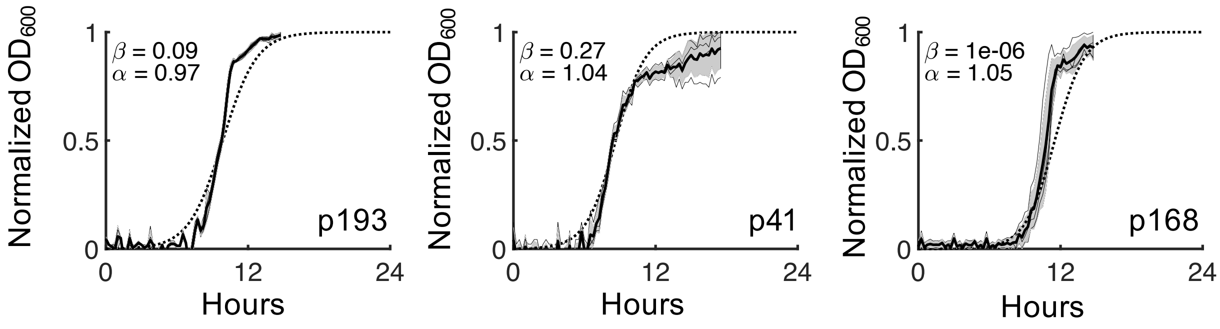

Appendix Figure S7: Fitted acquisition costs for diverse plasmids

Four plasmids are shown from left to right: RP4 (in this study), and p193, p41, and p168 (from previous work). Dark line shows average of three biological replicates. β was calculated by fitting data to our model and verified experimentally; ρ was fixed (0.35), and dotted lines show model fit using these values.

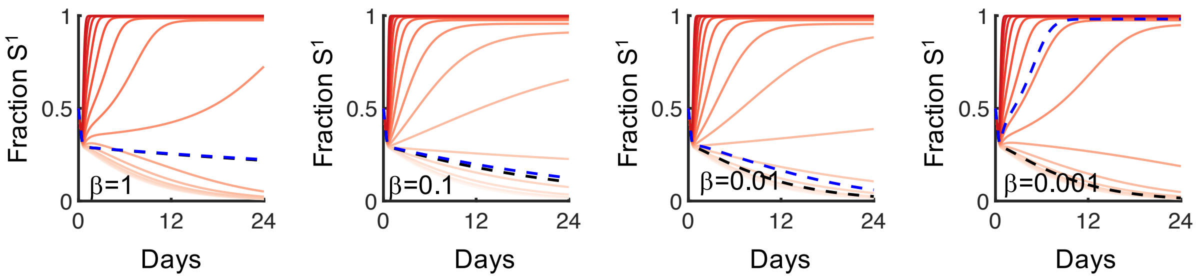

Appendix Figure S8: Updated persistence criteria

Increased acquisition cost skews 2-population predictions. Panels from left to right show S^1 fraction trajectories for increasing acquisition cost (corresponding to β decreasing from 1 to 0.001). In all panels, increasing values of η are shown in light to dark red; dashed black line corresponds to η_{crit} derived from the 2-population model ($\eta_{crit}=\alpha(\kappa+D)-D$), and blue dashed line corresponds to modified η_{crit} accounting for acquisition cost ($\eta_{crit}=(\alpha+\gamma/\beta)(\kappa+D)-D$), where $\gamma=0.001$ empirically approximates the magnification effect of β on α (e.g., when β is large the term is approximately equal to 0). The choice of γ does not qualitatively change these results: as acquisition cost increases, the 2-population prediction under-predicts the true η_{crit}; as a result, it incorrectly predicts that populations with intermediate conjugation efficiencies will persist when acquisition cost results in elimination (i.e. η values corresponding to trajectories that fall between black and blue dashed lines).

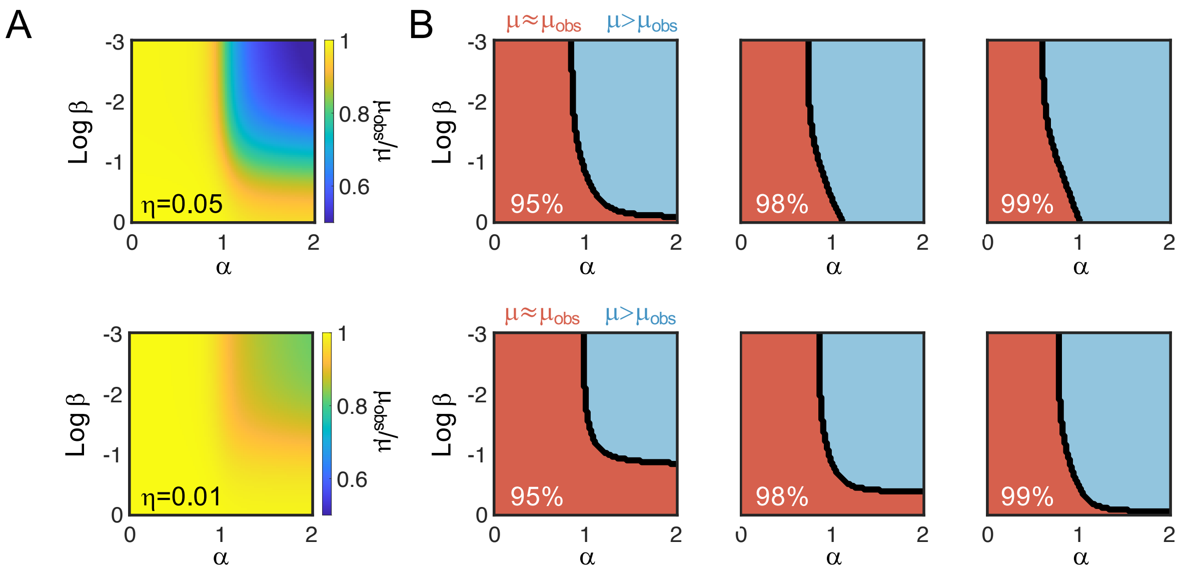

Appendix Figure S9: Growth rate ratio cutoff validation

A) The ratio between the maximum possible (μ) and observed (μ_{obs}) growth rate is calculated for a range of α and β; the top and bottom panels are heat maps for two representative conjugation efficiencies (η=0.05 or 0.01, respectively).

B) The boundary for a significant difference between the growth rates is calculated numerically based on whether the ratio from (A) is less than a certain percentage of the maximum (in this case equal to 1). The qualitative trends do not depend on this cutoff value.

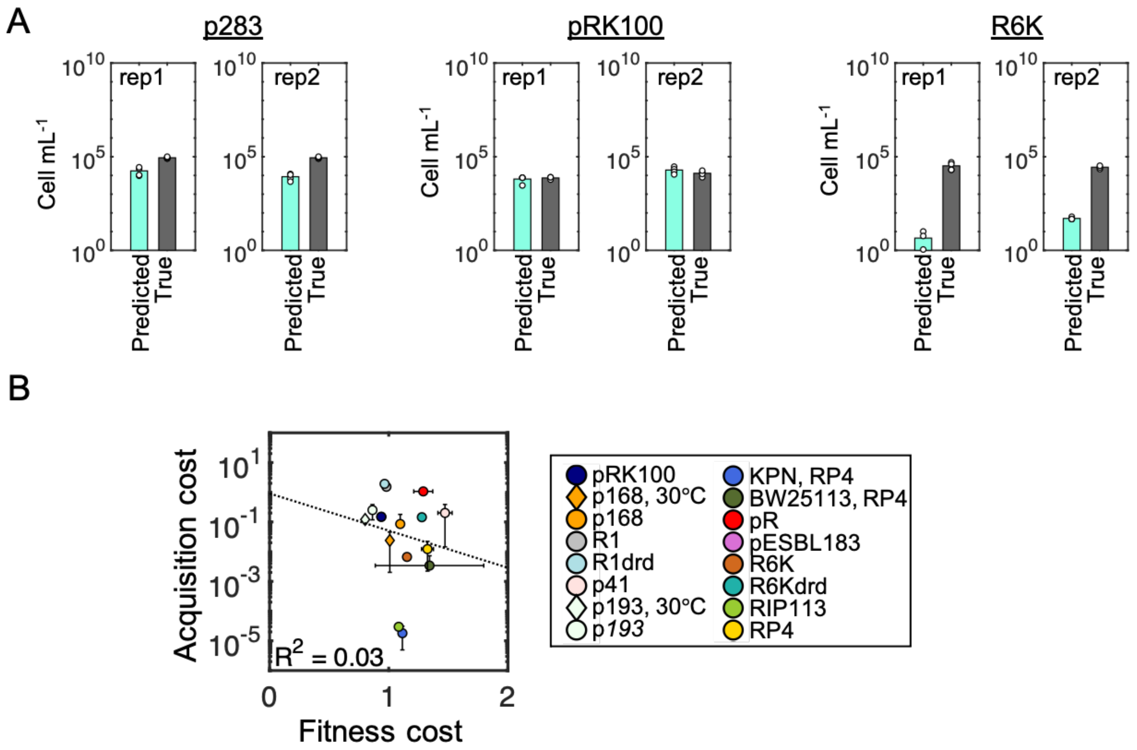

Appendix Figure S10: Data reproducibility

A) Biological replicates of acquisition cost measurements shown for representative plasmids. Plasmids were chosen to represent those with moderate (left), no (middle), or high (right) acquisition costs.

B) The relationship between fitness and acquisition cost was re-generated using averages of duplicate experiments whenever applicable, rather than the average of technical replicates. Doing so does not alter the R^2 value significantly (0.03 compared to 0.02). Error bars are only included for measurements with more than two biological replicates.

Appendix Table S1: Strains and plasmids used in this study

A. Strains and plasmids used in this study

Name	Strain Genotype	Plasmid	Antibiotic resistance*	Source
D	DA32838 Eco galK::cat-J23101-dTomato	RP4 (incP)	Kan, Cm	(Lopatkin *et al*, 2016)
R	*E. coli* MG1655 (K-12 F⁻ λ⁻ *ilvG⁻ rfb-50 rph-1*)	pCDF	Spec	(Lopatkin *et al*, 2016)
T	*E. coli* MG1655 (K-12 F⁻ λ⁻ *ilvG⁻ rfb-50 rph-1*); pCDF	pCDF, RP4	Spec, Kan	(Lopatkin *et al*, 2016)
pR donor	DA26735 Eco lacIZYA::FRT, galK::mTagBFP2-amp	pR, F_{hr} (incF)	Tet, Cm	(Lopatkin *et al*, 2016)
DA838F	DA32838 Eco galK::cat-J23101-dTomato	F_{hr}	Cm, Tet	(Lopatkin *et al*, 2016)
DA838	DA32838 Eco galK::cat-J23101-dTomato	N/A	Cm	(Lopatkin *et al*, 2016)
p41 donor	*E. coli* isolate number 41	p41 (incN/incF)	Carb, Amp, Ctx	(Lopatkin *et al*, 2016)
p168 donor	*E. coli* isolate number 168	p168 (incF/incN)	Carb, Amp, Ctx	(Lopatkin *et al*, 2016)
p193 donor	*E. coli* isolate number 193	p193 (incI/incF)	Carb, Amp, Ctx	(Lopatkin *et al*, 2016)
P (recipient for p41, p193, and p168)	*E. coli* MG1655 (K-12 F⁻ λ⁻ *ilvG⁻ rfb-50 rph-1*)	pTet_mCherry	Cm	Lab stock
KPN recipient	*Klebsiella pneumoniae* isolate KP0064, ST17	N/A	Cm	(Gomez-Simmonds *et al*, 2015)
p283 donor	ESBL 242	p283 (incI)	Carb	(Händel *et al*, 2015)
R6K donor	*E. coli* C600	R6K (incX)	Carb	(Lopatkin *et al*, 2016)
R6Kdrd donor	*E. coli* Dh5a	R6Kdrd (incX)	Carb	Generous gift from D. Mazel (Baharoglu *et al*, 2010)
R1 donor	*E. coli* MG1655 Δara	R1 (incF)	Kan	Generous gift from F. Dionisio and J. Alves Gama (Gama *et al*, 2020)
R1drd donor	*E. coli* MG1655 Δara	R1drd (incF)	Kan	Generous gift from F. Dionisio and J. Alves Gama (Gama *et al*, 2020)
pRK100 donor	*E. coli* HB101	pRK100 (incF)	Carb	Generous gift from T. Sysoeva
RIP113 donor	*E. coli* Dh5a	RIP113 (incN)	Tet	Generous gift from D. Mazel (Baharoglu *et al*, 2010)
RB933 recipient	*E. coli lacIZYA::scar galK::cat-YFP ΔgatZ::FRT-aph-FRT* $rpoB^{H526Y}$	N/A	Rif	Generous gift from I. Gordo (Leónidas Cardoso *et al*, 2020)

* Antibiotic abbreviations: Chloramphenicol (Cm); Kanamycin (Kan); Spectinomycin (Spec); Ampicillin (Amp); Carbenicillin (Carb); Tetracycline (Tet); Rifampicin (Rif); Ceftriaxone (Ctx)

B. Plasmid and recipient pairs used in this study

Plasmid	Recipient	Drug combination for selection (plasmid / recipient)
RP4	R	Kan (50 μg/mL) / Spec (50 μg/mL)
pR	DA838F	Kan (50 μg/mL) / Cm (100 μg/mL)
p41	P	Carb (100 μg/mL) / Cm (100 μg/mL)
p193	P	Carb (100 μg/mL) / Cm (100 μg/mL)
p168	P	Carb (100 μg/mL) / Cm (100 μg/mL)
p283	DA838	Carb (100 μg/mL) / Cm (100 μg/mL)
R6K	R	Carb (100 μg/mL) / Spec (50 μg/mL)
R6Kdrd	R	Carb (100 μg/mL) / Spec (50 μg/mL)
R1	RB933	Kan (50 μg/mL) / Rif (50 μg/mL)
R1drd	RB933	Kan (50 μg/mL) / Rif (50 μg/mL)
pRK100	R	Carb (100 μg/mL) / Spec (50 μg/mL)
RIP113	R	Tet (15 μg/mL) / Spec (50 μg/mL)

Appendix Table S2: Growth rate and lag time statistics using one-way ANOVA with Bonferroni correction

Method	Metric	Comparison	P-value
Baranyi	growth rate	plasmid-free, *de novo*	7.272E-09
Baranyi	growth rate	adapted, *de novo*	1.119E-08
Baranyi	lag time	plasmid-free, *de novo*	3.768E-09
Baranyi	lag time	adapted, *de novo*	5.711E-08
Baranyi	geometric lag time	plasmid-free, *de novo*	7.272E-09
Baranyi	geometric lag time	adapted, *de novo*	1.119E-08
Gompertz	growth rate	plasmid-free, *de novo*	1.028E-08
Gompertz	growth rate	adapted, *de novo*	1.291E-07
Gompertz	lag time	plasmid-free, *de novo*	6.510E-12
Gompertz	lag time	adapted, *de novo*	8.689E-10
Gompertz	geometric lag time	plasmid-free, *de novo*	4.303E-12
Gompertz	geometric lag time	adapted, *de novo*	7.608E-10
Logistic	growth rate	plasmid-free, *de novo*	4.411E-09
Logistic	growth rate	adapted, *de novo*	4.835E-08
Logistic	lag time	plasmid-free, *de novo*	6.966E-12
Logistic	lag time	adapted, *de novo*	8.115E-10
Logistic	geometric lag time	plasmid-free, *de novo*	5.992E-12
Logistic	geometric lag time	adapted, *de novo*	6.126E-10
Prensky	growth rate	plasmid-free, *de novo*	7.659E-09
Prensky	growth rate	adapted, *de novo*	1.839E-09
Prensky	lag time	plasmid-free, *de novo*	1.587E-08
Prensky	lag time	adapted, *de novo*	9.056E-08

Appendix Table S3: P-values for acquisition costs

A. One-tailed t-test for acquisition costs of all plasmids tested. Maximum p-value shown for RP4. Technical replicates were used for statistics in cases where only two biological replicates were measured due to low day-to-day variability (see Methods: Generality experiments).

Plasmid	P-value	Statistics replicate type	Biological replicates measured
RP4	0.0143	Biological	6
pR	0.3415	Biological	3
p41	7.0967e-05	Biological	4
p193, 30°C	2.8032e-04	Biological	3
p193, 37°C	0.0212	Biological	3
p168, 30°C	2.7185e-04	Biological	4
p168, 37°C	2.1021e-05	Biological	3
p283, 37°C	1.8984e-05	Technical	2
RIP113	6.7856e-05	Technical	2
pRK100	0.2768	Technical	2
R1	0.9331	Technical	3
R1drd	0.7939	Technical	3
R6K	7.5741e-05	Technical	3
R6Kdrd	0.0366	Technical	3

B. One-tailed t-test for acquisition costs under altered glucose/casamino acid conditions. In all cases, biological triplicates were used.

Plasmid	Condition 1	Condition 2	p-value
RP4	Glucose=0.4, CAA=0	Glucose=0.4, CAA=0.01	4.1833e-04
RP4	Glucose=0.4, CAA=0	Glucose=0.4, CAA=0.1	7.8656e-05
RP4	Glucose=0.4, CAA=0.01	Glucose=0.4, CAA=0.1	0.0517
RP4	Glucose=0.4, CAA=0.01	Glucose=0.04, CAA=0.1	0.0387

Appendix Table S4: Model parameters and variable values

Parameter	Description	Value
μ	Maximal specific growth rate	0.7 hr-1
α	Fitness cost	[0-2]
D	Dilution rate	0.006 hr^{-1}
ρ	Growth rate inhibition	[0-1]
β	Transition rate	[0-1] hr^{-1}
κ	Plasmid segregation error rate	0.001 hr^{-1}
S^A	Plasmid-carrying adapted population	[0-1e-3]
S^D	Plasmid-carrying *de novo* population	[0-1e-3]
S^0	Plasmid-free population	[0-1e-3]
t	Simulation time	[0:24] for short term, [0:504] for long term

References

Baharoglu Z, Bikard D & Mazel D (2010) Conjugative DNA transfer induces the bacterial SOS response and promotes antibiotic resistance development through integron activation. *PLoS Genet* 6: 1–10

Gama JA, Fredheim EGA, Cléon F, Reis AM, Zilhão R & Dionisio F (2020) Dominance Between Plasmids Determines the Extent of Biofilm Formation. *Front Microbiol* 11: 2070

Gomez-Simmonds A, Greenman M, Sullivan SB, Tanner JP, Sowash MG, Whittier S & Uhlemann AC (2015) Population structure of Klebsiella pneumoniae causing bloodstream infections at a New York City tertiary care hospital: Diversification of multidrug-resistant isolates. *J Clin Microbiol* 53: 2060–2067

Händel N, Otte S, Jonker M, Brul S & ter Kuile BH (2015) Factors That Affect Transfer of the IncI1 $β$-Lactam Resistance Plasmid pESBL-283 between E. coli Strains. *PLoS One* 10

Leónidas Cardoso L, Durão P, Amicone M & Gordo I (2020) Dysbiosis individualizes the fitness effect of antibiotic resistance in the mammalian gut. *Nat Ecol Evol* 4: 1268–1278

Lopatkin AJ, Huang S, Smith RP, Srimani JK, Sysoeva TA, Bewick S, Karig DK & You L (2016) Antibiotics as a selective driver for conjugation dynamics. *Nat Microbiol* 1: 16044
